# Morphology and morphometry of the inner ear of the dromedary camel and their influence on the efficiency of hearing and equilibrium

**DOI:** 10.1186/s40851-022-00196-0

**Published:** 2022-10-27

**Authors:** Safwat Ali, Abdelraheem Esmat, Atef Erasha, Masahiro Yasuda, Mohamed Alsafy

**Affiliations:** 1 Department of Anatomy and Embryology, Faculty of Veterinary Medicine, Minia University, Minia, Egypt; 2 Department of Anatomy and Embryology, Faculty of Veterinary Medicine, University of Sadat, Sadat City, Egypt; 3 Department of Veterinary Anatomy, Faculty of Agriculture, University of Miyazaki, Miyazaki, Japan; 4 Department of Anatomy and Embryology, Faculty of Veterinary Medicine, Alexandria University, Abees 10th, Alexandria, Egypt

**Keywords:** Camel, Inner ear, Gross morphology, Computed tomography, Endocast

## Abstract

**Background:**

The inner ear morphology and size are linked to hearing and balance ability. The goal of this study was to determine the morphology and morphometrics of the dromedary camel's inner ear and how it influences hearing accommodation and equilibrium in the desert environment.

**Materials and methods:**

Gross morphology, computed tomography images, and the endocast were used to show the inner ear morphology. A caliper and ImageJ software were used to take measurements on a plastic endocast.

**Results:**

The presence of the subarcuate fossa, flat cochlea, radii curvature of the semicircular canals, particularly the lateral semicircular canal, orthogonality, and the union between the semicircular canals, along with slightly increased saccule and utricle size, maintains camel balance on sandy ground, even during heavy sandstorms. The cochlear basilar membrane length and cochlea radii ratio aided low-frequency hearing and perception over a wide octave range.

**Conclusion:**

The camel's cochlear characteristics revealed a lengthy basilar membrane, a high radii ratio, 3.0 cochlear canal turns, and a very broad cochlea. The orthogonality of the semicircular canals, the high curvature of the lateral semicircular canal, the presence of the subarcuate fossa, and the confluence between the lateral and posterior semicircular canal were particular specifications that allowed the inner ear of the camel to adapt to desert living.

## Introduction

Dromedary camels (*Camelus dromedarius*) live in the desert and hot climates. The physical aspects of sound propagation, such as spreading and frequency- and humidity-related attenuation, demand the awareness of specific frequencies in those difficult environmental conditions. Abiotic noise, particularly wind, limits auditory awareness over a narrow frequency range [1].

In the desert, hearing requires challenging sound spreading in this special type of nature, for the attenuation caused by humidity reflects on the frequency. Also, abiotic noise by wind forces the animals’ inner ears to perceive in a low zone of frequencies. These conditions need sensitivity at a particular level of frequencies [2–4]. The camel is a significant livestock species uniquely acclimatized to hot arid environments. For survival in the desert, the camel possesses physiological, behavioral, and anatomical adaptation mechanisms. Despite the large body mass of the camel and the nature of the desert land with the force of sandstorms, it seems that the camel has no problem maintaining a balanced movement which has motivated many researchers in biotechnology, genetics, and physiology to understand the biology of the camel [2–4].

The camel was used for transportation, agricultural work, and military expeditions. These activities necessitate the camel's remarkable agility, especially given its massive body bulk, uneven terrain, and regular high sand storms [2–4].

Estimating hearing abilities from gross cochlear dimensions and shape has been utilized for many years [5, 6], in particular to measure hearing abilities in fossil and extant mammal ears that cannot be researched experimentally. The sensitivity of the inner ear to head rotation and locomotor behaviors is related to the semicircular canal diameters and orientations [7].

In ground-dwelling mammals, the number of spiral turns in the cochlea is proportional to the octave range of audible frequencies [8]. The length of the basilar membrane is inversely related to the hearing's high and low-frequency limits [8]. In addition, the higher the low-frequency sensitivity, the greater the radii ratio of the spiral turns. According to the energy density concentrating theory [9], this association represents a functional anatomical correlation. The width of the cochlea and the width of the whorl of the basal turn are also two of the most critical adaptations that aid in the perception of very high frequencies. Cochlear width was found to be strongly positively linked with the high-frequency limit and, as a result, the optimal hearing frequency [10].

The semicircular canal's sensitivity is related to the radius of curvature of the semicircular canals. Larger semicircular canals are more responsive in animals of identical body proportions [11]. In addition, various physiological studies estimated and reported deviations of semicircular canals from the orthogonal designs of the skull [12]. The vestibular sensitivity and variability of the semicircular canals from orthogonality have a negative relationship [13].

The subarcuate fossa is a depression located caudal and lateral to the internal acoustic meatus [14]. It houses the petrosal lobule of the cerebellar paraflocculus and is absent in many mammals [15]. The paraflocculus is involved in motor coordination. As a result, it was hypothesized that the cerebellar paraflocculus’s big petrosal lobule and subarcuate fossa are associated with complex motor coordination [16].

The cochlear shape is linked somehow to the camel’s agility and capacity for balance. Previous research found that there is a positive relationship between the cochleae width and the camel speed: the cochlea of quick taxa is broader than those of slow taxa. The camel cochleae are broad, which gives them their flat shape. We suggested from our previous findings and prior theory that camel could be placed among fast animals [17].

The membranous labyrinth contains two enlargements in the center, which are known as the utricle and saccule. Three semicircular ducts arise from the utricle, which is responsible for balance, and the spiral cochlear duct originates from the saccule, which is responsible for hearing [18]. Meanwhile, it has been shown that the saccule is involved in assistance of the function of the cochlea in difficult hearing conditions and takes part in the perception of high-intensity, low-frequency tone [19].

Therefore, this study aimed to evaluate the morphology and morphometrics of the osseous labyrinth of the camel's inner ear and determine how much it can affect the animal's capacity to adapt to the desert environment in terms of hearing and balance.

## Materials and methods

### Animals

Seven heads of healthy dromedary camels (*Camelus dromedarius*) of varying ages (2.5 to 5.5 years old) were collected from a local slaughterhouse in Minia Governate, Egypt (these animals were slaughtered for meat purposes under expert veterinarian supervision). These samples were used to identify the morphology and perform the measurements of the inner ear (Table 1).

**Table 1:** The number of samples, the age of animals, and the technique used

			**Sample**	**Age**
**The cochlea**			3 Endocast	3 years
Basilar membrane		mm		
The radius of curvature	Base	-		
	Apex	-		
Radii ratio		-		
**The semicircular canal**				
The anterior semicircular canal radius of curvature				
The posterior semicircular canal radius of curvature				
The lateral semicircular canal radius of curvature				
The average a semicircular canal radius of curvature				
Cochlear width		mm	2 Gross anatomy	4.5 – 5
Cochlear height		mm		
Cochlea shape index		-		

### Gross morphology

Two heads were used for the anatomical dissection. They were cut in a sagittal plane that allowed the study of the petrous part of the folloculonoduolar lobe of the cerebellum. The whole brain was removed to study the shape and structure of the internal acoustic meatus and the subarcuate fossa.

The petrous bone was extracted as a whole by dissection starting from the ramus of the mandible cranially, the paracondylar process caudally as the dorsal border, 1 cm above the internal acoustic meatus. After that, the mastoidectomy was performed as it is performed in humans, and the bone between the external ear canal's caudal wall and the temporal line was removed gradually. This procedure was continued until entry to the middle ear cavity became possible.

### Computed aid tomography scans (CAT)

Two heads were used for computed aid tomography scans within 2 h after they were collected. Serial transverse CT bone window scans were used on the head from the external occipital protuberance caudally to the level of the orbital rim rostrally to evaluate the ear parts related to the bone landmark levels. The scanning conditions were: 120 kV and 150 mA; the window width and level (W/L) were 1126/213 using a Hitachi CAT (CT-W450-10A, Hitachi, Japan).

### Endocast

For the measurement and anatomical analysis of the cochlea and semicircular canals, three heads were used for the making of a 3D plastic endocast model. Six casts were photographed under a stereomicroscope using a fine-scale ruler and caliper. The polyester resin was injected in wax through one window and closed in the other. After hardening the polyester resin, the bone was dissolved with nitric acid (5%) for 10 days. When the bone became jellylike, the bone was removed.

### Measurement methods

**Fig. 1:**
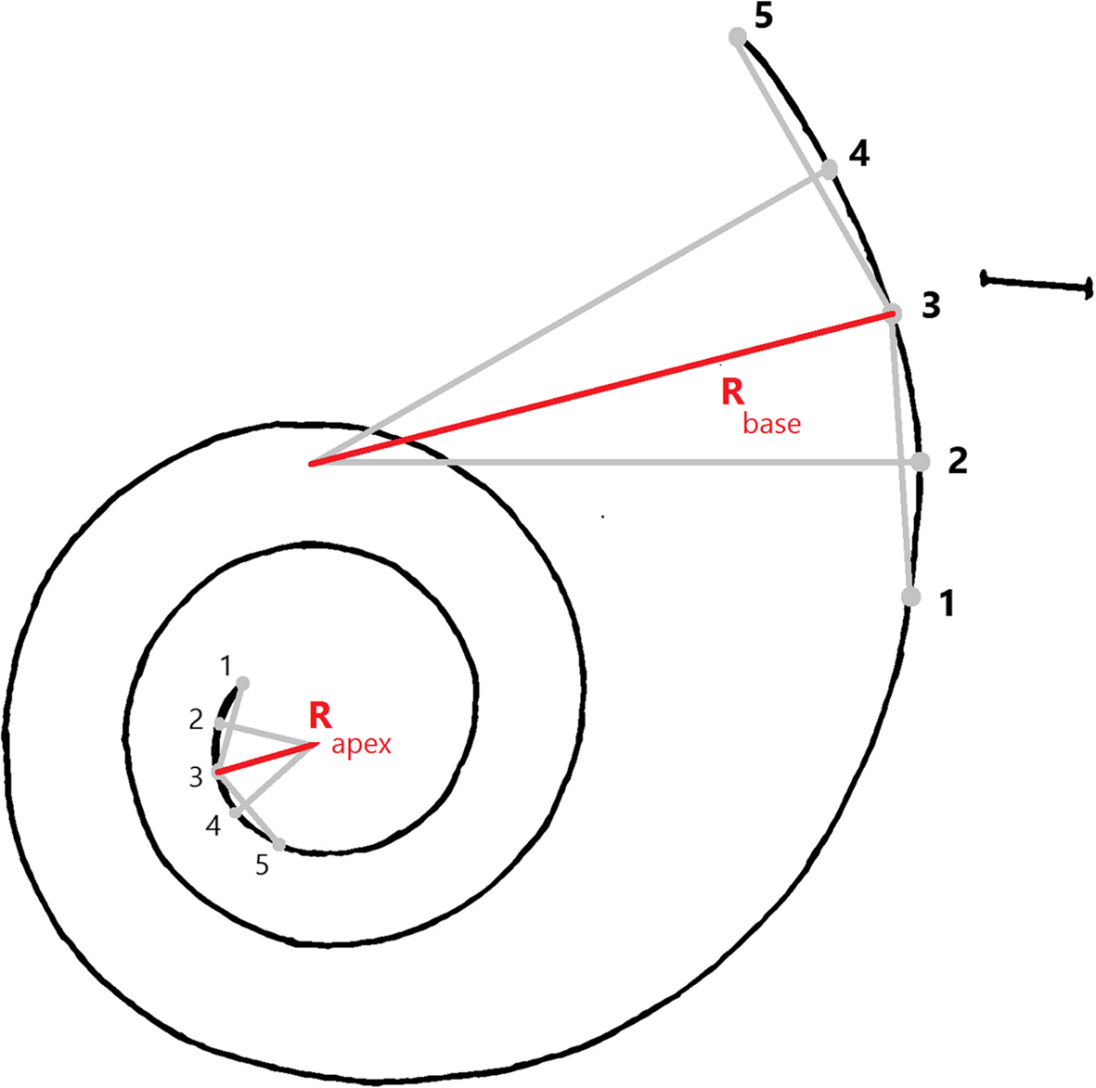
Schematic of the cochlear spiral of the camel with five points used to determine radii of curvature. Spiral Radii were calculated as follows: Five equally spaced points 1–5 were chosen on each of the first and last quarter turns of the estimated basilar membrane paths. The first point was chosen just apical to the cochlear hook. Two chords were constructed between points 1 and 3 and 3 and 5. Perpendiculars to the chords were constructed through points 2 and 4. The intersection of the two perpendiculars determined an area center of curvature from which the radius was decided

**Fig. 2:**
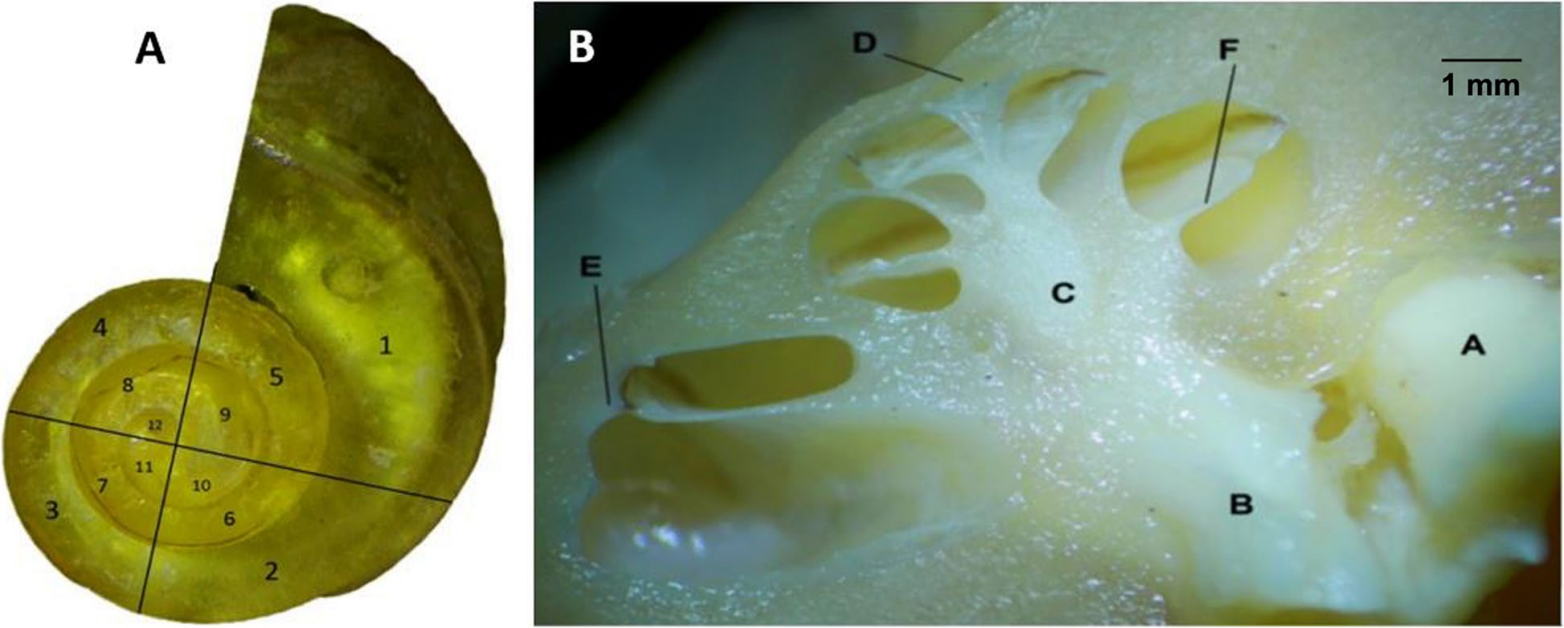
**A**. Plastic cast of the camel labyrinth showing the spiral turns of the cochlea as viewed from the apex. The number of quarter-turns within the spiral might be counted by counting the number of times the spiral path intersected the two-plane containing the projection line and therefore the central axis of the spiral. **B** The cross-section in the camel cochlea after decalcification shows the height and width of the cochlea. A. Facial nerve, B. Cochlear nerve, C. Modiolus, D. Helicotrema, E. Spiral ligament, F. Secondary lamina

The measurements were taken on a plastic endocast using a caliper and ImageJ software. The cochlear spiral turns were calculated following the protocols of [8]. By drawing, a line starting from the round window passes through the central axis. The number of half-turns within the spiral was counted by counting the number of times the spiral path intersected the plane containing the projection line and the central axis of the cochlear turns (Figs. 1, 2). The degree of coiling exhibited by the cochlea is reported (in degrees) alongside the number of completed turns (calculated as total degrees divided by 360°). By using another perpendicular line, the number of turns can also be counted by dividing the sum of quarter turns by four. A shape index (aspect ratio) of the cochlear spiral was calculated by dividing the peak of the spiral by the width of the basal turn. A high ratio was that above 0.55, and flattened cochleae had a ratio of 0.55 and below [20]. The total length of the cochlear canal was measured using ImageJ software. The length of the cochlear canal approximated the length of the soft tissue of the basilar membrane [21]. Spiral Radii were calculated as follows: Five equally spaced points 1–5 were chosen on each of the first and last quarter turns of the estimated basilar membrane paths. The first point was chosen just apical to the cochlear hook. Two chords were constructed between points 1 and 3 and 3 and 5. Perpendiculars to the chords were constructed through points 2 and 4. The intersection of the two perpendiculars determined an area center of curvature from which the radius was decided [9] (Fig. 1).

**Fig. 3:**
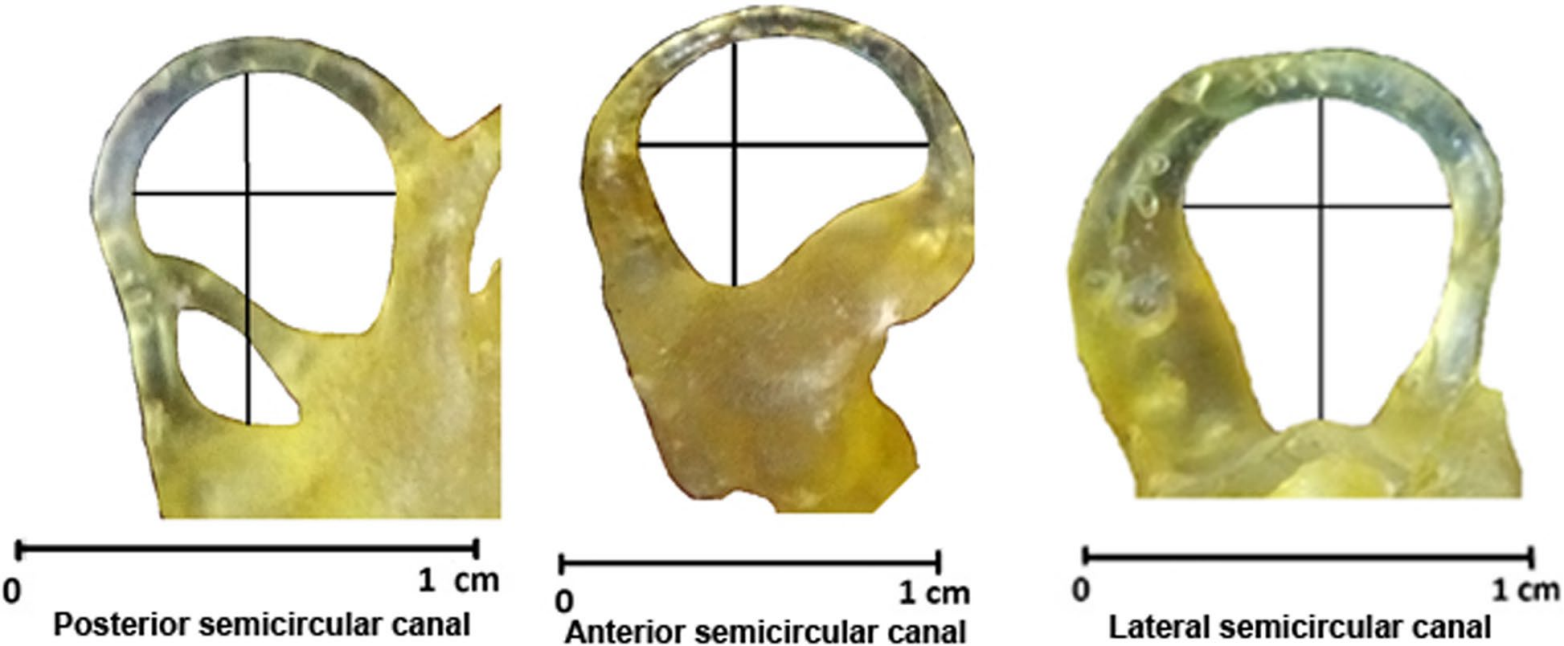
Endocast with drawn lines showing how the measurements for the calculation of radius of curvature of each semicircular canal were taken. The radius of curvature of each semicircular canal is calculated as half the average between the height and width of the canal arc

Linear measurements were typically examined for the inner ear [22]. The radius of curvature of each semicircular canal was calculated as half the average between the height and width of the canal arc (Fig. 3). The arc radius of a canal was half the average of the height and width of the arc. The height of the anterior and lateral semicircular canals was calculated as the greatest distance perpendicular to the plane of the lateral canal from the vestibule wall to the center of the lumen of the canal. From the center of the lumen of the common crus to the center of the lumen of the canal’s caudal limb, the height of the posterior semicircular canal was measured parallel to the plane of the lateral canal. The greatest distance from the vestibule wall to the center of the canal’s lumen was used to calculate the height of the posterior semicircular canal.

The nomenclature was according to NAV [23].

## Results

Anatomically, the inner ear consists of the osseous labyrinth and membranous labyrinth.

### Osseous labyrinth

**Fig. 4:**
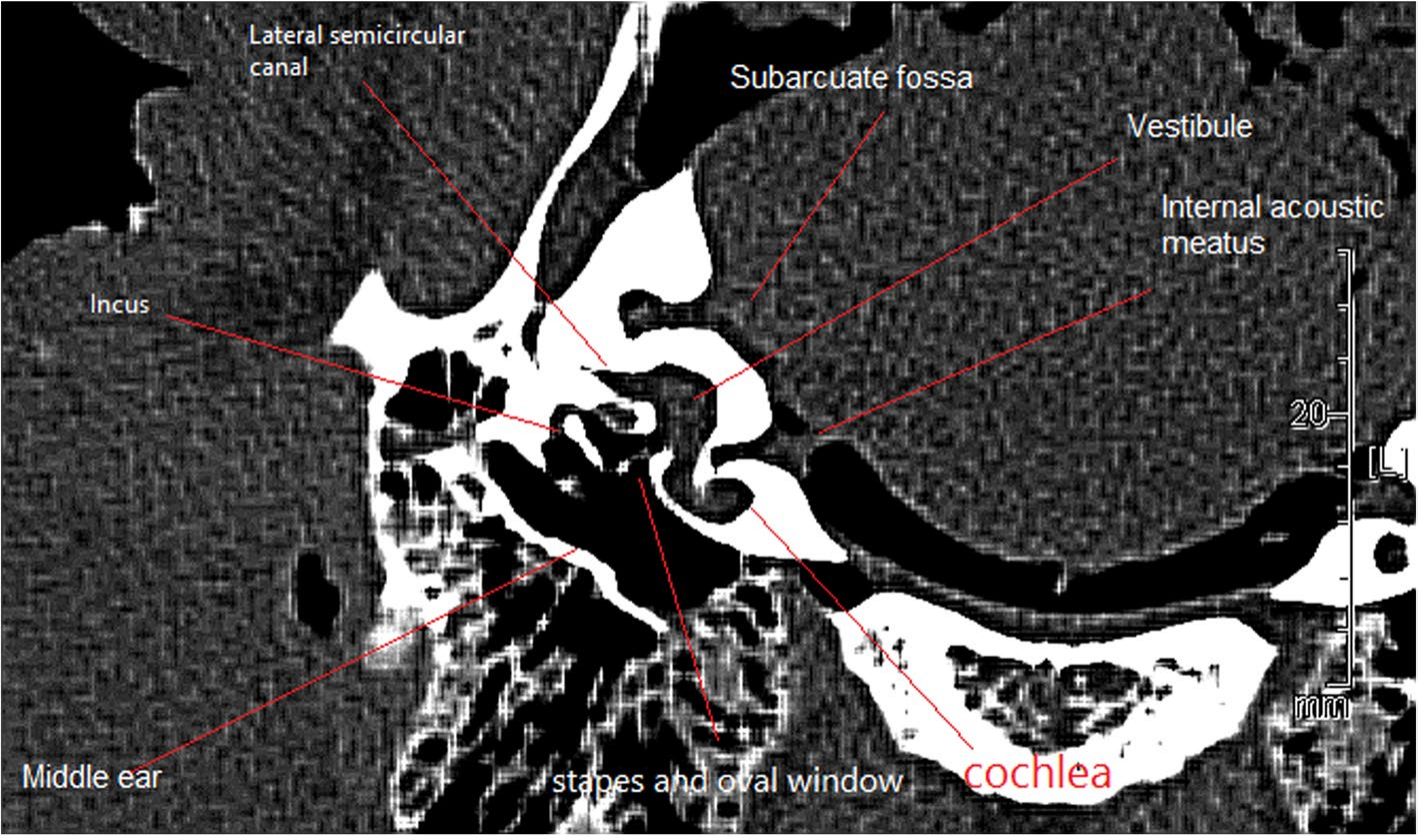
Transverse computed tomography image of the camel temporal bone at the level of the left lateral semicircular canal

**Fig. 5:**
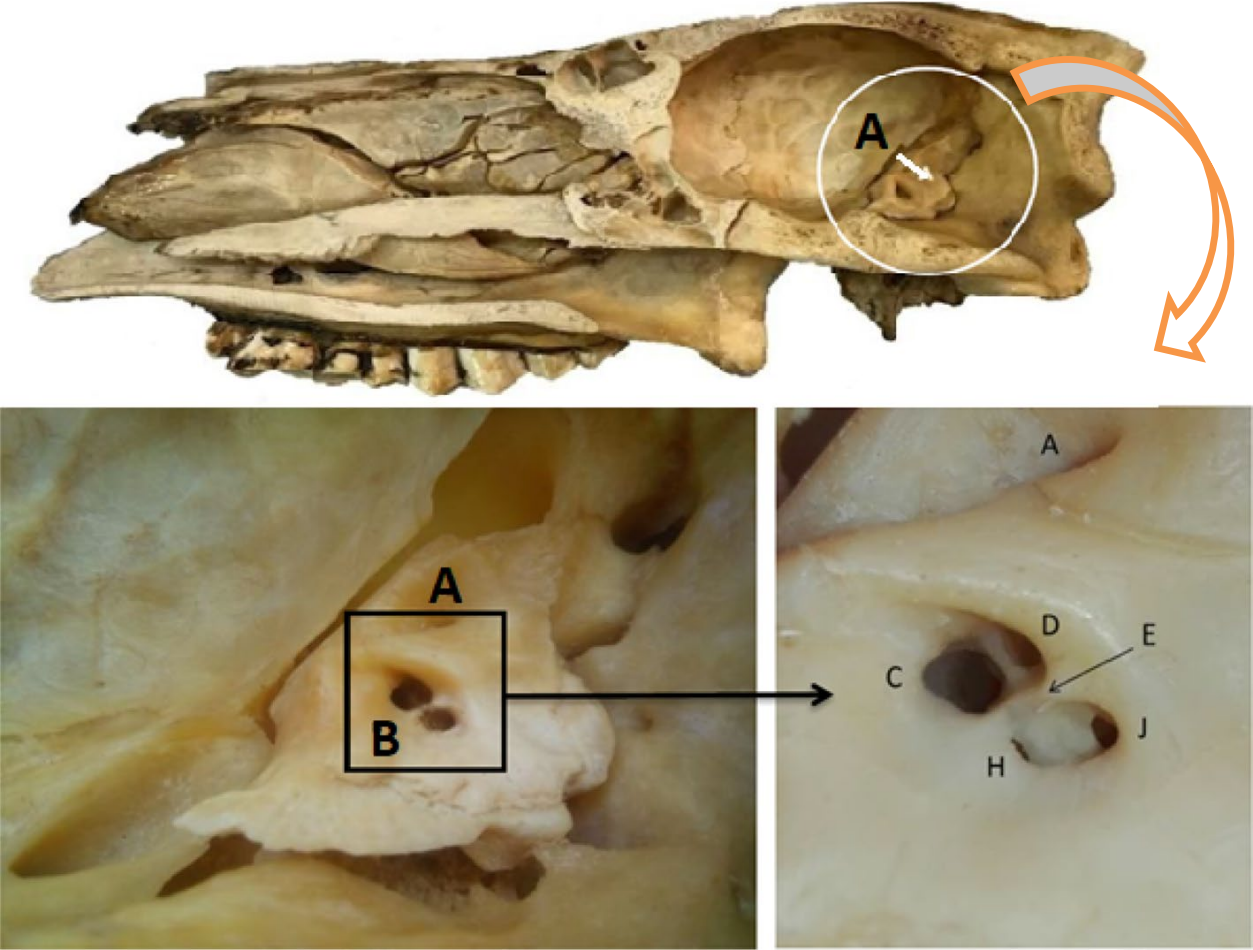
The sagittal section in the camel skull. A. Petrosal bone and gross morphological image of the camel internal acoustic meatus. A. Subarcuate fossa, B. Internal acoustic meatus, C. Facial nerve foramen, D. Foramen for utricle and the ampullae of the anterior SC nerve, H. Foramen for cochlear nerve, J. Foramen for saccule and the ampulla of the posterior semicircular canal, E. Ridge dividing the upper and lower part of the internal acoustic meatus

**Fig. 6:**
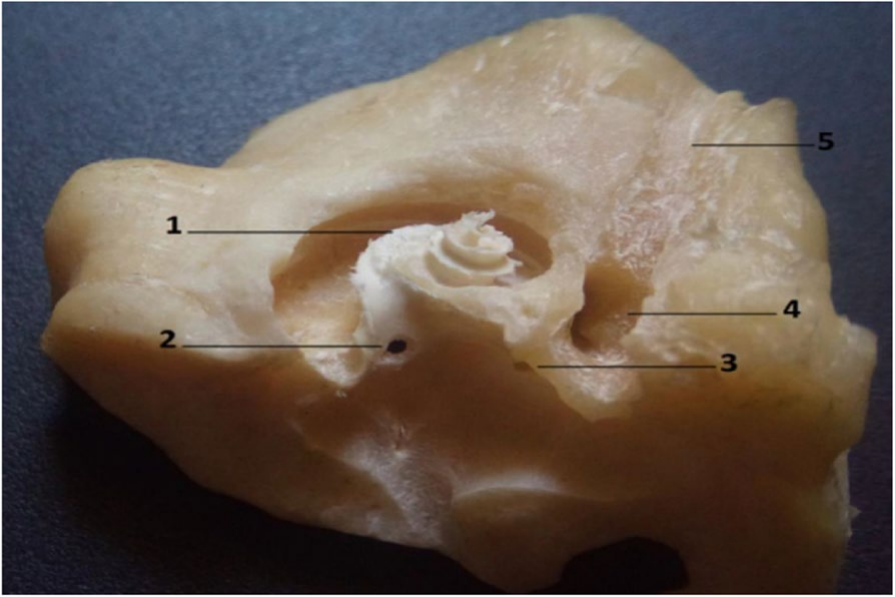
Gross morphological image of bony specimen shows the middle ear side of inner ear after uncovering of the cochlea of the camel. 1. Cochlea, 2. Foramen for the saccular part of the vestibular nerve, 3. Foramen for utricle part of the vestibular nerve, 4. Facial canal, 5. Canal for greater petrosal nerve

The osseous labyrinth consisted of a series of cavities within the petrous part of the temporal bone (Figs. 4, 5, and 6) that included the vestibule (Fig. 4), semicircular canals (Fig. 4), and the cochlear canal (Figs. 4, 5), which communicated with the vestibule via the internal acoustic meatus (Figs. 4, 6).

#### Cerebellar surface of the petrous part of the temporal bone

The inner ear was separated from the cranial bone, and its cerebellar surface was relatively concave and characterized by two large depressions (Fig. 5/A). The first was the shallow internal acoustic meatus (Fig. 5/B), which was caudal and ventral overlying the cochlea. The second depression was the subarcuate fossa, which was lateral and caudal to the internal acoustic meatus (Figs. 4; 5/A).

The internal acoustic meatus was located on the cerebellar surface of the petrous part of the temporal bone and acted as a link between the cranial cavity and the inner ear (Fig. 5/B). At the bottom, at the fundus of the internal acoustic meatus, four foramina for facial and vestibulocochlear nerves were detected (Figs. 5/C,H; 6/2,3). A clear transverse crest appeared oblique, caudal, and dorsal, which divided these four foramina into two groups: two placed dorsal and rostral, and two placed ventral and caudal (Fig. 5/E). At the restoral foramen of the dorsal foramina, the facial nerve passes through the mastoid part of the temporal bone (Figs. 2B/A; 5/C; 6/4), which is the largest. The other foramen was situated caudally to the utricle and the ampulla of the anterior SC nerve (Figs. 5/D; 6/2,3). The cochlear nerve passed through the restoral foramen of the ventral foramina (Figs. 2B/B; 5/H) and the other foramen, which was situated caudal to the ampulla of the posterior semicircular duct nerve (Fig. 5/J). A canal for the greater petrosal nerve was detected (Fig. 6/5).

### Cochlea

**Fig. 7:**
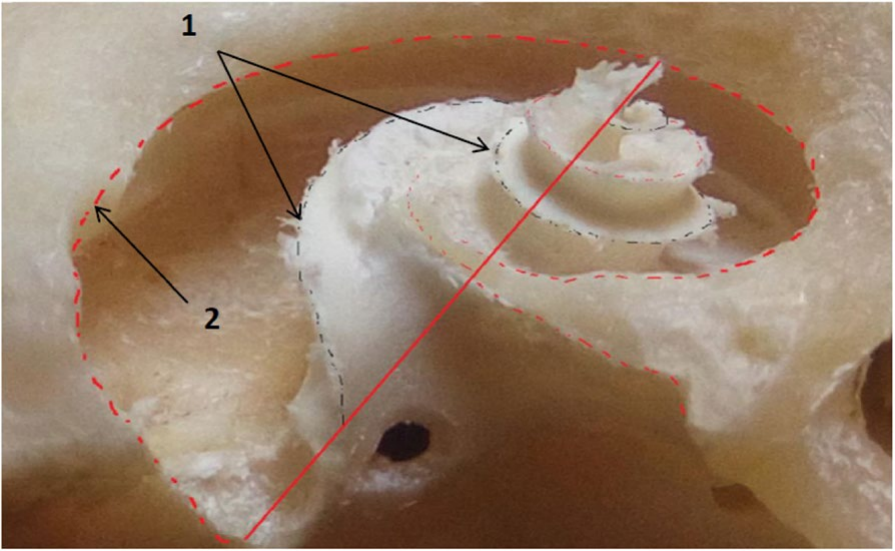
Gross morphological image of the camel cochlea. 1. Primary spiral lamina, 2. Secondary spiral lamina

The cochlea was a bony cone positioned within the petrous part of the temporal bone; it consisted of the osseous cochlea surrounding the cochlear membranous duct (Fig. 6/1), which was formed spirally upward around a central column of the bone, the modiolus. The modiolus had a thin bony spiral lamina (Fig. 2B/C). The primary and secondary osseous spiral lamina were located in the petrous part of the temporal bone (Fig. 7/1, 2). The primary osseous spiral lamina was present throughout the basal turn (Fig. 7/1), extended to the entire cochlear length, and projected from the cochlear canal’s inner wall. The secondary osseous spiral lamina was projected from the cochlear canal outer wall (Fig. 2B/D, E).

The length of the basilar membrane was 40.5 mm. The radius of curvature of the camel cochlea at the base was 3.894, the radius at the apex was 0.429, and the radii ratio was 9 (Figs. 1; 2A). The cochlea number of turns was about three turns, a rotation of 1080° (Fig. 2A). The cochlear width at the lowest cochlear turn was 11 mm, the height was 6 mm, and the cochlea shape index was 0.55; these parameters made the cochlea of the camel flat type cochlea (Fig. 2B). The diameters of the whorl and the tube of the cochlea in front of the round window were 4 mm.

### Semicircular canals

**Fig. 8:**
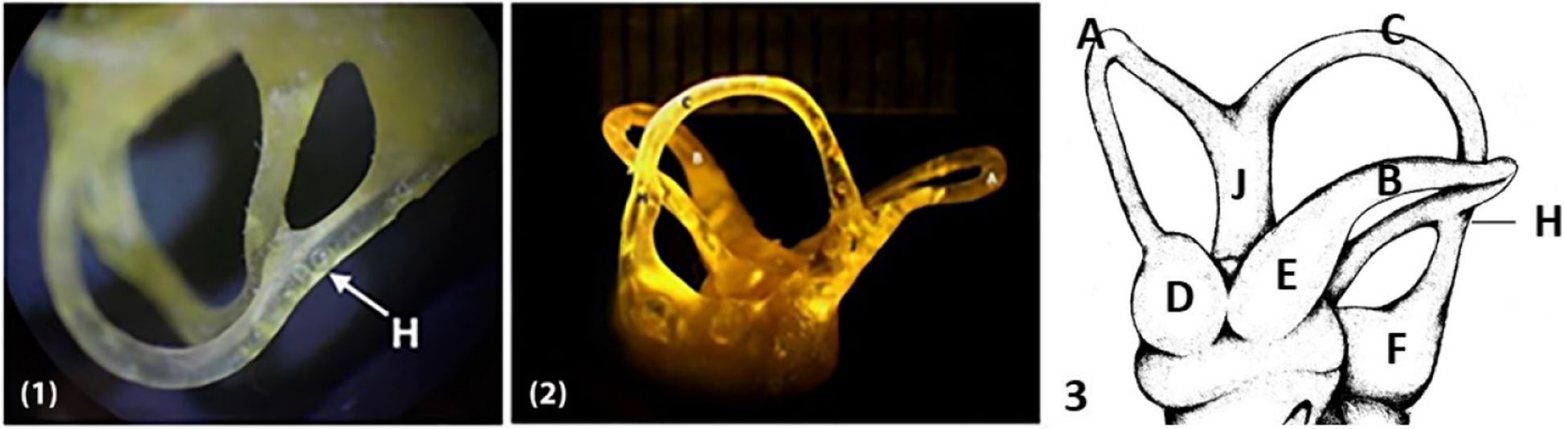
(1 and 2) Corrosion cast from plastic material of a camel semicircular canal. (3) Diagram showing the shape of the three semicircular canals of the camel. A. anterior semicircular canal, B. lateral semicircular canal, C. Posterior semicircular canal, D. ampulla of anterior semicircular canal, E. ampulla of lateral semicircular canal, F. ampulla of posterior semicircular canal, H. Confluence between posterior and lateral semicircular canal, J. common crus

The camel had three semicircular canals: the anterior, lateral, and posterior (Figs. 8/A, B, C; 8/2, 3, 4), which were located in the petrous part of the temporal bone. Each semicircular canal formed two-thirds of a circle. The anterior semicircular canal had the most circular overall shape, the lateral semicircular canal was nearly circular or irregular, and the posterior semicircular canal was oblong (Figs. 3/A, B, C; 8/1, 2, 3).

This study showed the orthogonality of the three semicircular canals (Fig. 8/1, 2, 3). There were limited specimens for this measurement, so the variation in the same species needs more investigation. These canals are approximately 90 degrees apart. The anterior duct was oriented transversely, the caudal duct sagittally, and the lateral duct horizontally. In the same horizontal plane, the anterior and lateral ampullas connect to the vestibule (Fig. 8/D, E). However, the caudal ampulla is located significantly below or ventral to the common crus (Fig. 8/F, J). The radius of curvature of the anterior semicircular canal was 4.75, that of the posterior canal was 4.13, and that of the lateral canal was 3.63. The average radius of curvature of a semicircular canal was 4.17. The average radius of curvature of a semicircular canal was 4.17.

**Fig. 9:**
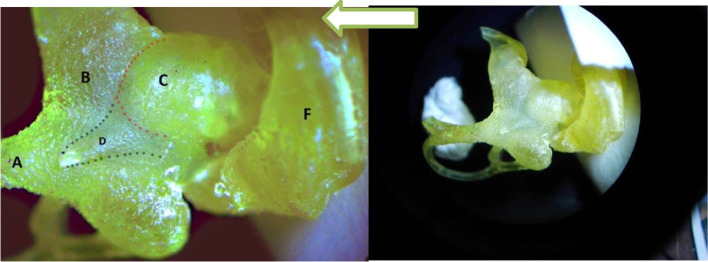
A corrosion cast from plastic material of the camel inner ear. A. Common crus, B. Elliptical recess, C. Spherical recess, D. Vestibular aqueduct, F. Basal turn of the cochlea

A confluence appeared between the caudal arm of the lateral semicircular canal and the inferior arm of the posterior semicircular canal (Fig. 8/H). This confluence did not result in a secondary crus commune where the two semicircular canals became close. The anterior semicircular canal was fitted to the outer restoral edge of the fossa (Fig. 8/2) and was directed laterally, and the fossa extended through the anterior semicircular canal into the mastoid and vestibular labyrinth regions of the petrosal bone. The vestibule occupied a relatively large volume in the labyrinthine cavity (Fig. 4). Although the relation between bony and membranous size is not 1:1, a large utricle and saccule were indicated in proportion with a large elliptical and spherical recess (Fig. 9/A, B, C, D).

### Subarcuate fossa

The volume of the subarcuate fossa has measured the endocast approximated with the skull size, where our study found that the camel had a large-sized subarcuate fossa (Figs. 4; 5/A; 10/1; 11A). This fossa was located at the petrosal lobule of the cerebellar paraflocculus (Figs. 4; 11B). This fossa was situated above the internal acoustic meatus (Fig. 5/B) and separated from the latter by a raised bony ridge-like area (Fig. 5/E).

**Fig. 10:**
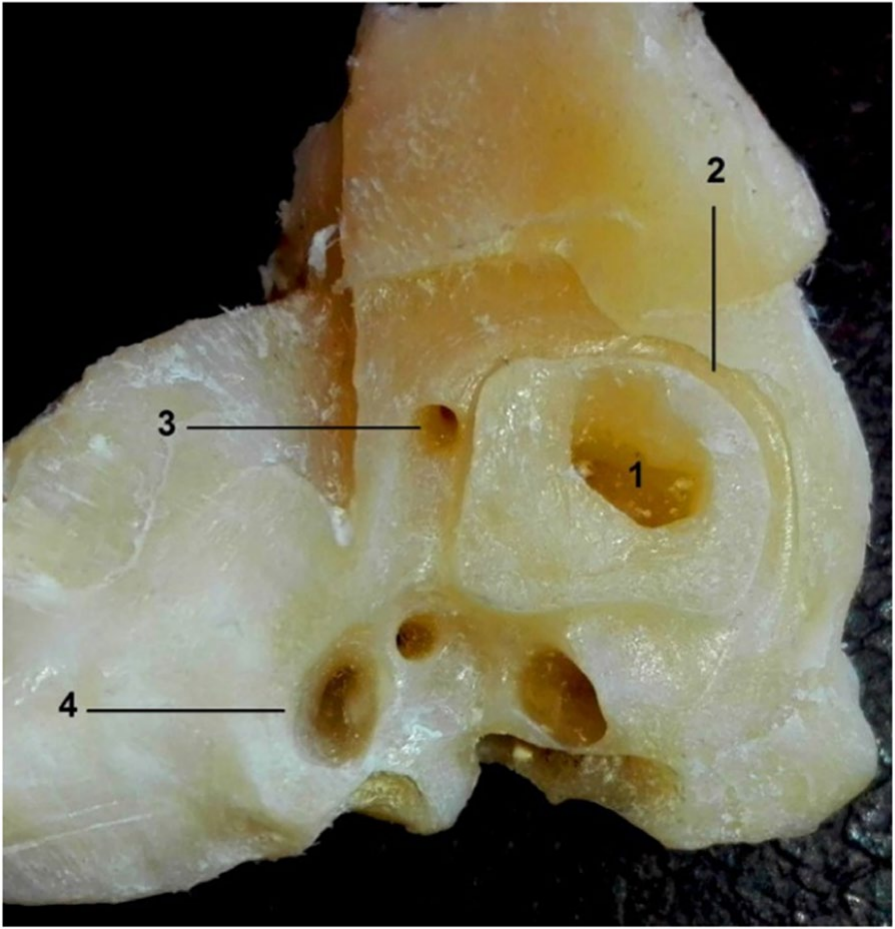
A sagittal section in the semicircular canal parallels the anterior semicircular canal. 1. Subarcuate fossa, 2. Anterior semicircular canal, 3. Posterior semicircular canal, 4. Lateral semicircular canal

**Fig. 11:**
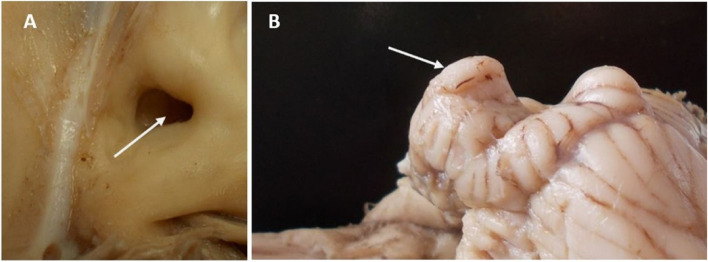
Gross morphological images show the subarcuate fossa and involved part of the cerebellum. A. arrow shows the subarcuate fossa in the fresh sample. B. arrow shows the petrosal lobule of the cerebellar paraflocculus

### Discussion

The cochlea, vestibule, and three semicircular canals make up the inner ear of animals. The cochlea is responsible for hearing, while the vestibule and three semicircular canals are responsible for balance [7]. Although the camel shares the same inner ear outline structure and perception as other mammals, there are differences in the parameters compared to those of the other species. The camel's auditory and locomotor physiology is comparable to other mammals [7].

The length, number of turns, and curvatures of the cochlear spiral affect the efficacy of hearing in animals [8, 9, 24, 25]. Taking body size into account, the camel’s cochlea has a higher score in those parameters than the ground-dwelling mammals studied in previous studies [8, 9]. Therefore, those parameters help the camel to cope with sound propagation that characterizes the desert environment, such as spreading and frequency- and humidity-related attenuation of abiotic noise.

There is a strong relationship between basilar membrane length and high—and low-frequency hearing limits for different species. The ability to perceive at a low frequency is necessary due to the physical characteristics of sound propagation and abiotic noise in the desert [1]. Long basilar membranes have been linked to a decrease in audible frequencies [8, 26]. A high radii ratio improves low-frequency sensitivity [9]. With a basilar membrane length of 40.5 mm, the camel has high measures of both the basilar membrane and radii ratio among various animals, compared to the cow’s 38 mm and the cat’s 22–23 mm [8]. Camel’s radii ratio is 9, cat’s is 6.2, cow’s 8.9, and human’s 8.2. With the characteristics of the desert, this morphometrics qualifies the camel for high perception at a low-frequency of demand [1].

The current study noted that, in comparison to other animals, the camel has 3.0 cochlear turns with a rotation of 1080°; carnivores have 3.0 turns and horses have 2.5 turns, pigs have 4.0 turns, and ruminants have 3.5 turns [27]. The camel's cochlear coil degree has been rated as high, which helps to increase its octave range of hearing [8]. We contend that this satisfies the camel’s need to distinguish between wide ranges of pitches to deal with the abiotic cacophony of the desert. The cochlear width of the camel was 11 mm, compared to 7.5 mm for sheep and 5.5 mm for calves [28].

Like every mammal, the camel has anterior, lateral, and posterior semicircular canals [29]. The function of these canals is the sense of balance [18], and there is a sizable variation in their size among animals associated with functional variations in locomotion [30]. This study supports this hypothesized association by relating the camel’s semicircular canal shape to the need for movement in the environment and comparing the results to those of other species.

Due to the force of sandstorms and its large body mass, it is difficult for the camel to maintain its balance of movement in the desert. We can provide information on the camel's speed, vestibular sensitivity, and equilibrium based on the measurement of the radius of curvature of the semicircular canals performed in this study.

The correlation between agility and the lateral semicircular canal was the lowest. The camel’s lateral semicircular canals have a radius of curvature of 3.63 mm. It is the largest in animals, as evidenced by the sizes in horses (3.23 mm), cows (2.35 mm), gorillas (3.05 mm) [31], bovines (2.15 mm), and cats (1.39 mm) [32]. Additionally, the camel’s semicircular canals are oriented at 90 degrees to one another, enabling the vestibular system to function at its best [13]. The radius of curvature of the semicircular canals has been linked to canal sensitivity and animal agility [11].

The junction of the inferior arm of the posterior canal with the caudal arm of the lateral canal was a notable characteristic of the camel semicircular canals. Until now, the role of this union was unknown. This phenomenon, called semicircular canal dehiscence, can be present in humans. Normally, this condition is not present in extant animals but it exists in extinct mammals, and therefore researchers hypothesized that the bony confluence of the area mentioned above is accompanied by a confluence also in membranous ducts based on bony specimens available [33]. The development of a secondary crus commune is linked to the all other living mammals [34–36]. However, the camel's confluence does not produce a secondary crus commune. It was hypothesized that this feature in the camel is supported by a unique form of equilibrioception that is not present in any other living mammals.

The current study found that the camel is distinguished from other ruminants and large mammals by its large subarcuate fossa. This fossa housed the cerebellum's folloculonoduolar lobe, which is thought to be a component of the “vestibulocerebellum” concept [37]. As a consequence, more complex motor coordination may be associated with the large subarcuate fossa and a larger folloculonoduolar lobe of the cerebellum [16]. Furthermore, the semicircular canal size may be affected by the subarcuate fossa, which is located between the canals [38, 39].

The cochlea of the camel is believed to be flat, as indicated by the cochlear shape index [20], with an aspect ratio implicated in the animal’s agility, and in fast taxa, the cochleae are broader than those of slow taxa [17].

In challenging hearing situations, the saccule aids the cochlea’s function and can contribute to the perception of loud, low-frequency tones [19]. In light of this, a camel’s big saccule indicates a highly sensitive hearing perception. These findings are supported by the relatively large size of the vestibule and the subsequently big size of the saccule.

## Conclusion

The inner ear of the camel is constructed in a particular way to facilitate life in the desert. Significant dimensions of the cochlear parameters allow the camel to hear at low frequency and over a large octave range while adapting to the physical characteristics of sound transmission and avoiding abiotic noise. The camel was found here to have an extremely broad cochlea, a long basilar membrane, a high radii ratio, and 3.0 turns of the cochlear canals, all of which were indicators of this. The orthogonality of the semicircular canals, the high curvature of the lateral semicircular canal, the presence of the subarcuate fossa, and the confluence of the lateral and posterior semicircular canals could all contribute to the camel’s ability to traverse desert terrain.

## Data Availability

The datasets used and/or analyzed during the current study are available from the corresponding author on reasonable request.
